# The Hospital Outlook for 1910

**Published:** 1910-01-01

**Authors:** 


### THE HOSPITAL OUTLOOK FOR 1910.

With genuine satisfaction we wish every hospital worker everywhere a bright and prosperous New Year. At no period in the history of the voluntary hospitals of this country has the outlook been brighter than it is to-day. As we showed last week, notwithstanding the assertions of a few croakers, who persist in maintaining falsely that the voluntary system is breaking down, and that the voluntary hospitals are without adequate funds, the exact contrary is the fact. Wherever a voluntary hospital nowadays falls behind financially, it is generally possible to prove on investigation that the fault lies not with the voluntary system but with the managers responsible for the drooping hospital who have failed to exhibit sufficient enterprise and energy in making the claims of their institution known to the public. In no single instance where the managers of a voluntary hospital have resorted to modern methods and spread their net wide enough, have they failed to reap a revenue from all sources equal to the needs of their institutions. With the exception of half a dozen hospitals in London, where the causes of the drooping are well known and remediable, financially speaking the voluntary hospitals were never more healthy than they are at this moment. The same is true of the voluntary hospitals throughout the country. This splendid result testifies to the high average of efficiency which is now the rule at our voluntary hospitals. Where funds are lacking and large deficiencies have accumulated the evil arises mainly from the fact that the managers have been foolish enough not to exert themselves to wipe out each year's deficiency as, and when, it occurs. We hope, therefore, that the public, in apportioning their gifts to hospitals, will continue to show that they look into the actual condition and working of each hospital before sending a contribution, and that they will make the largest gifts to those hospitals where the management takes care to keep the expenditure well within the resources available each year.

The result of the inspections of the principal voluntary hospitals and Poor-Law infirmaries, to which work Sir Henry Burdett has devoted so much time and thought, is another encouraging feature in the outlook for 1910. With the exception of less than a dozen institutions, the average of efficiency is already very high, and, where the standard has not yet been reached, the committee has welcomed the criticisms which have been made, and has resolutely set to work to bring the institution up to the required standard. At Exeter and Plymouth much discussion has arisen in consequence of the publication of Sir Henry's reports, and at the present time the committees of these institutions are busily engaged in concerting measures whereby we hope everything possible will be done to make these institutions in all respects modern and up-to-date hospitals. In several centres schemes are under consideration for rebuilding and reconstruction, involving large sums of money, though the hospital buildings have become altogether out of date, and though the truest economy will be to scrap the buildings and erect in their place new and up-to-date hospitals upon a novel plan of construction which will reduce the cost per bed from £700 or £800, to which it has grown in recent times, to something like £300 or less. In this connection we are glad to know that in one great city, where all the hospitals are out of date and require rebuilding, the citizens are considering whether or not it may be possible to set up a Hospital City outside the urban boundaries, or well away from the contaminating atmosphere of the town, and to erect entirely new buildings of the type just mentioned, whereby the cost of administration will be materially reduced, the efficiency of treatment raised, and the average residence of each acute case diminished. The first great town which adopts the Hospital City idea will become a modern Mecca for the sick, to which the whole world is likely to be attracted in order to see how each great urban population may provide adequately for its sick at the smallest possible cost in the best possible way. When once this idea has taken practical shape it is likely to become speedily the plan which will be followed in due course by every important city throughout the civilised world.

